# Quantitative Evaluation of the Relationship between T-Wave-Based Features and Serum Potassium Level in Real-World Clinical Practice

**DOI:** 10.1155/2018/3054316

**Published:** 2018-12-18

**Authors:** Dukyong Yoon, Hong Seok Lim, Jong Cheol Jeong, Tae Young Kim, Jung-gu Choi, Jong-Hwan Jang, Eugene Jeong, Chan Min Park

**Affiliations:** ^1^Department of Biomedical Informatics, Ajou University School of Medicine, Suwon, Gyeonggi-do, Republic of Korea; ^2^Department of Biomedical Sciences, Ajou University Graduate School of Medicine, Suwon, Gyeonggi-do, Republic of Korea; ^3^Department of Cardiology, Ajou University School of Medicine, Suwon, Gyeonggi-do, Republic of Korea; ^4^Department of Nephrology, Ajou University School of Medicine, Suwon, Gyeonggi-do, Republic of Korea

## Abstract

**Background:**

Proper management of hyperkalemia that leads to fatal cardiac arrhythmia has become more important because of the increased prevalence of hyperkalemia-prone diseases. Although T-wave changes in hyperkalemia are well known, their usefulness is debatable. We evaluated how well T-wave-based features of electrocardiograms (ECGs) are correlated with estimated serum potassium levels using ECG data from real-world clinical practice.

**Methods:**

We collected ECGs from a local ECG repository (MUSE™) from 1994 to 2017 and extracted the ECG waveforms. Of about 1 million reports, 124,238 were conducted within 5 minutes before or after blood collection for serum potassium estimation. We randomly selected 500 ECGs and two evaluators measured the amplitude (T-amp) and right slope of the T-wave (T-right slope) on five lead waveforms (V3, V4, V5, V6, and II). Linear correlations of T-amp, T-right slope, and their normalized feature (T-norm) with serum potassium levels were evaluated using Pearson correlation coefficient analysis.

**Results:**

Pearson correlation coefficients for T-wave-based features with serum potassium between the two evaluators were 0.99 for T-amp and 0.97 for T-right slope. The coefficient for the association between T-amp, T-right slope, and T-norm, and serum potassium ranged from -0.22 to 0.02. In the normal ECG subgroup (normal ECG or otherwise normal ECG), there was no correlation between T-wave-based features and serum potassium level.

**Conclusions:**

T-wave-based features were not correlated with serum potassium level, and their use in real clinical practice is currently limited.

## 1. Introduction

Hyperkalemia is an electrolyte derangement that can lead to fatal cardiac arrhythmia. Proper management of hyperkalemia has become more important because of the increased prevalence of hyperkalemia-prone diseases, such as diabetes mellitus, coronary artery disease, and chronic kidney disease [1]. Hyperkalemia and hypokalemia or fluctuations in potassium levels are associated with an increased risk of mortality and life-threatening arrhythmias [2–6]. Moreover, morbidity, hospitalization, and death can follow even minor changes in potassium level in patients with renal or cardiac disease [1].

Many of the key drugs used for disease treatment alter serum potassium levels. Medications targeted at the renin-angiotensin-aldosterone system have been the mainstay of treatment for cardiovascular disease or for the prevention of chronic kidney disease progression. The Eighth Joint National Committee guidelines recommend aldosterone receptor blockers as key drugs for secondary prevention of heart failure because aldosterone antagonists can reduce mortality due to heart failure [7, 8]. However, it is ironic that the use of aldosterone antagonists increases mortality due to hyperkalemia, which emphasizes the importance of proper management of hyperkalemia [9]. Non-steroidal anti-inflammatory drugs are other medications that cause severe hyperkalemia but are administered without proper electrolyte level monitoring. In addition, other risks are associated with potassium-rich foods, which can often be fatal in patients with end-stage renal disease.

Alterations in electrocardiogram (ECG) patterns are known to be directly associated with serum potassium levels [10, 11]. Mild to moderate hyperkalemia can lead to PR interval prolongation and the development of peak T-waves. Severe hyperkalemia can cause the QRS complex to widen. Flattened or inverted T-waves, a U wave, ST depression, and a wide PR interval are observed in patients with hypokalemia. Because of prolonged ventricular repolarization, a prominent U wave occurs, or a prolonged QT interval can be observed when U waves are superimposed on a T-wave.

Changes in ECG patterns due to an elevated potassium level are clear in the experimental setting. However, many studies have reported that these patterns are not reliable clinically [12]. Some previous studies have reported that the performance of potassium level estimation using ECG information by physicians was poor. The sensitivities of hyperkalemia detection by two physicians were 0.43 and 0.34 [13]; even when subjects had moderate to severe hyperkalemia (potassium level >6.5 mmol/L), the sensitivities were only 0.62 and 0.55. According to another retrospective review, T-wave changes assessed by a cardiologist were also not well correlated with serum potassium level, and most T-wave changes were nonspecific [12].

Based on known T-wave patterns, however, other studies have attempted to determine potassium levels using machine learning [14–16]. According to these studies involving patients undergoing hemodialysis, single-lead ECG data (V3, V4, or V5) are as precise as 12-lead ECG data with reported absolute errors of 0.5±0.42 and 0.46±0.39 mmol/L [15, 16], respectively. These studies suggest that ECG patterns, especially the shape of the T-wave, could be helpful in determining serum potassium levels in clinical settings. However, these studies have the limitation that the model that was employed was developed and validated with a limited number of patients (26 patients for the development and 19 for the validation), and the subjects were restricted to patients on hemodialysis.

Moreover, to the best of our knowledge, no study has directly evaluated how well T-wave-based features correlate with serum potassium level in the real clinical practice setting. In the present study, we conducted quantitative evaluation of ECGs captured in real-world clinical practice to determine whether T-wave-based features are useful for estimating serum potassium level in general clinical practice.

## 2. Methods

The requirement for informed consent was waived and the study was approved by the Ajou University Hospital Institutional Review Board (IRB) (IRB number AJIRB-MED-MDB-17-273). We only used de-identified data and analyzed the information retrospectively.

### 2.1. Data Source

**Figure 1:**
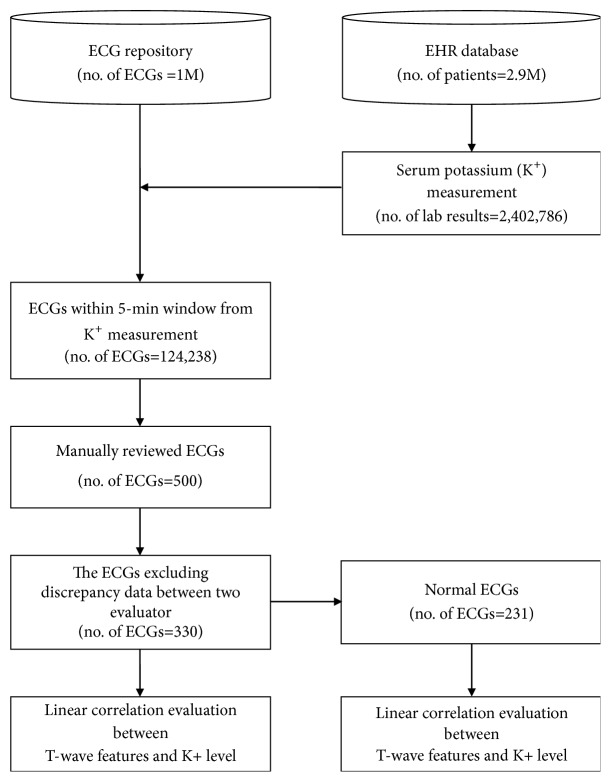
**Overview of the study process. **Three hundred and thirty manually reviewed electrocardiograms (ECGs) were used to evaluate the linear correlation between T-wave features and serum potassium level. Two hundred and thirty-one ECGs were independently analyzed to exclude bias due to abnormal heart rhythm. ECG: electrocardiogram; no.: number; M, million; EHR: electronic health record.

We used a clinical research database that included patient demographics, diagnoses, drug prescriptions, and laboratory test results extracted from the electronic health records of a tertiary teaching hospital in Korea (Ajou University Hospital) between September 1994 and December 2017 (Figure 1). The database included 134,011,566 prescriptions, 32,956,672 diagnoses, and 278,011,281 laboratory test results from 2,940,379 patients.

**Figure 2:**
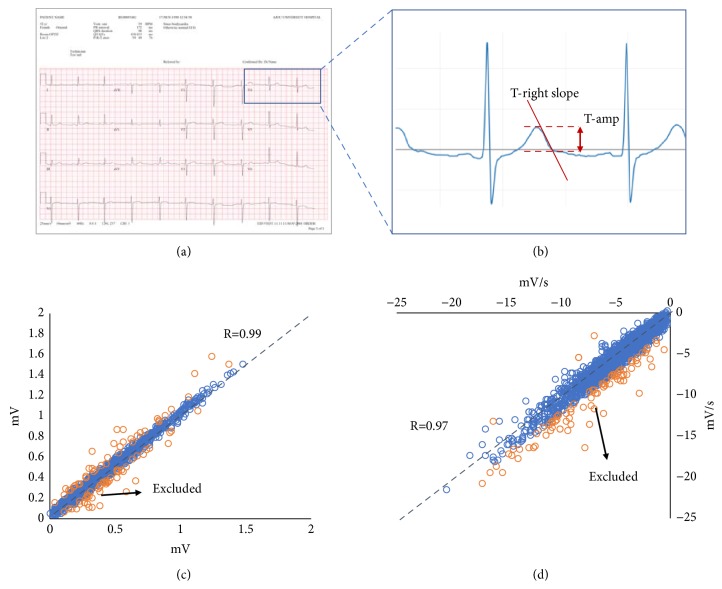
**Process and quality evaluation of T-amp and T-right slope measurements.** Original ECGs are stored in PDF format (a). ECG waveforms have been extracted and evaluated using web-based evaluation tools (b). Measurements of T-amp (c) and T-right slope (d) between the two evaluators are well correlated. Measurements that have a discrepancy between the two evaluators (marked with orange color) are excluded from further analysis. T-amp: amplitude; T-right slope: right slope of T-waves.

The ECG typically consists of alphanumeric values and waveform graphs (Figure 2(a)). Alphanumeric values included demographics, the patient identification number, date of electrocardiography, and ECG parameters (RR, QT intervals, etc.). Waveform graphs are time series data representing changes in electronic signals from the heart over a few seconds. After all data from the ECGs, which were stored in PDF format in the local ECG repository (MUSE™ system), were collected, the part containing the waveform was extracted and transformed to SVG format [17]. Subsequently, we converted the x- and y-coordinates of vector images into an equidistant time series (500 data points per second, 500 Hz) via linear interpolation to retain a data format similar to that obtained from the sensor directly.

Of about 1 million collected ECGs, 124,238 were obtained within 5 minutes (time window) before or after blood collection for serum potassium estimation. Of these, we randomly selected 500 ECGs for manual evaluation.

### 2.2. Data Preprocessing

A web-based tool was developed to measure amplitude (T-amp; the difference in millivolts (mV) between the peak and the end of the T-wave) and the right slope of the T-wave (T-right slope; the slope at the steepest part of the descending portion of the T wave). This tool helped us evaluate and efficiently manage the measurement results of each ECG signal (Figure 2(b)) quickly. The tool displays a 3-second ECG waveform, allowing the user to measure T-amp and T-right slope. Regarding the 500 selected ECGs, T-amp and T-right slope on the waveforms of five leads (V3, V4, V5, V6, and II) were manually and independently measured by two evaluators using this tool.

Waveforms in the ECGs usually included two or three beats. Evaluators selected the beat of the baseline that was most stable and had less noise. T-amp and T-right slope were measured on selected beats. Measurements of differences between the two evaluators that were greater than mean+2×standard deviation (SD) or less than mean–2×SD were excluded from further analysis (Figures 2(c) and 2(d)). The degree of correlation between the two evaluators was determined by Pearson correlation coefficient analysis.

### 2.3. Feature Extraction

We excluded ECGs, which had discrepancies in their interpretation between the two evaluators in one or more leads. T-amp and T-right slope values measured by the two evaluators were averaged and used as final values of T-amp and T-right slope of corresponding ECGs. According to the following formula, which was used to normalize features for estimating serum potassium level in a study by Zachi et al., [16] two features were normalized and integrated into one feature:

(1) $$\begin{array}{c} T-norm=\frac{T-right\;\;slope}{\sqrt{T-amp}} \end{array}$$

First, we measured or calculated the three features (T-amp, T-right slope, and T-norm) in each lead of the ECGs. Second, the lead that had the most prominent T-wave (the largest T-amp) among V3, V4, and V6, and II, named Pt, was selected and used as the representative feature of each ECG.

### 2.4. Feature Evaluation and Statistical Analysis

One-way analysis of variance and Post-Hoc Tukey's test were conducted to evaluate the difference between measured T-amp and T-right slope values between different leads. A* p*-value <0.05 was considered significant.

We evaluated the linear correlation between T-wave features and the actual serum potassium level using Pearson correlation coefficient analysis. To exclude the effect of underlying diseases, which can affect cardiac rhythm, we conducted subgroup analysis in which only ECG results showing normal ECG (n=191) or otherwise normal ECG (n=40)—the normal ECG subgroup—was included. The detailed interpretation lists of otherwise normal ECG and the count per interpretation are provided in Table S1.

MS-SQL 2017 (Microsoft Corp.) was used for data management, and R (version 3.2.2, Foundation for Statistical Computing) was used for data preprocessing and statistical analysis.

## 3. Results

### 3.1. Datasets for Analysis

T-amp and T-right slope from 500 ECGs were measured by two evaluators. The measurements between two evaluators were well correlated in terms of both T-amp and T-right, as shown in Figures 2(c) and 2(d). After excluding ECGs that were discrepant between the two evaluators, data from 330 ECGs (including 231 ECGs from the normal ECG subgroup) were finally selected. The baseline characteristics of the subjects are shown in Table 1.

Absolute values of measured T-amp and T-right slope were highest in lead V3 and lowest in lead II for both the total number of subjects (n=330) and for the normal ECG subgroup (n=231). The values were significantly higher in precordial lead than in lead II in both groups (*p*<0.001).

### 3.2. Linear Correlation between T-Wave-Based Features and the Serum Potassium Level

**Figure 3:**
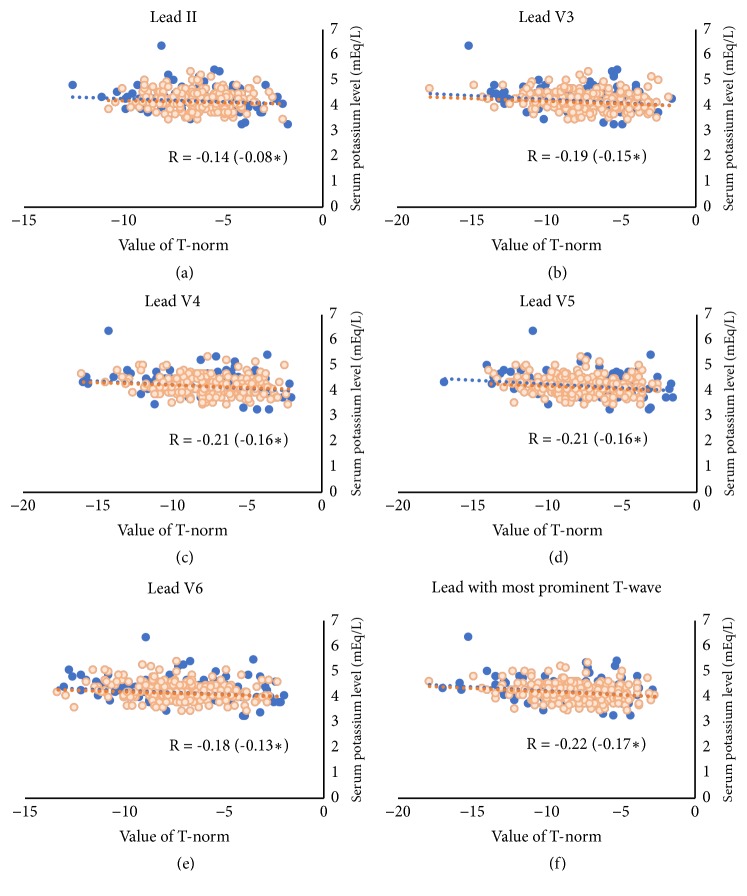
**Linear correlations between features of T-norm and serum potassium level.** There is no linear correlation in all leads: II (a), V3 (b), V4 (c), V5 (d), V6 (e), and the lead with the most prominent T-wave (f). Data of the normal ECG subgroup (normal ECG or otherwise normal ECG) is marked with orange color and their correlation coefficients are marked with ‘*∗*'. Absolutely no correlation was found in all leads. T-norm: normalized feature.

Pearson correlation coefficients of T-amp with the serum potassium level in all the leads indicated a positive correlation but the coefficient values were low and ranged from 0.08 to 0.19 (Table 2). In contrast, T-right slope and T-norm had a negative correlation but their coefficient values were also low (T-right slope: range -0.11 to -0.02; T-norm: range -0.22 to -0.14). Correlation degrees were generally the lowest at lead II and the highest at Pt, the lead that had the most prominent T-wave. However, Pt also showed a poor linear correlation with the serum potassium level (Figures 3(a)–3(f)). Lead V3 was mainly selected (53.3%) for Pt, followed by leads V5 (15.8%), V4 (13.9%), V6 (13.6%), and II (0.03%).

### 3.3. Linear Correlation in Normal ECG Subgroup

After excluding abnormal ECGs (only normal ECGs or otherwise normal ECGs), the results showed the same pattern as those from total number of ECGs selected; similar to the results in the total number of ECGs, the Pearson correlation coefficients of T-amp were positive and those of T-right slope and T-norm were negative in all leads. However, there was no correlation between T-wave-based features and serum potassium level in this subgroup as coefficient values ranged from -0.17 to 0.16 (Figure 3 and Table 2).

## 4. Discussion

This study directly evaluated the degree of correlation between blood potassium concentration and T-wave-based features of ECGs. Manually reviewed T-wave-based features of ECGs conducted in daily practice did not correlate with serum potassium level. Moreover, in the normal ECG subgroup, we did not detect any correlation.

In this study, the T-amp and T-right slope from the waveforms of five leads (V3, V4, V5, V6, and II) were selected and evaluated. The waveforms of the four leads (V3-V6) were used in previous studies [16] for estimating the serum potassium level. Lead II is most popularly used in patient monitoring. Thus, we aimed to evaluate the possibility of applying the features to a clinical setting where patients are monitored, such as in an intensive care unit.

The pattern of values, which was extracted as features (T-amp and T-right slope from lead II, V3, V4, V5, and V6), showed well-known patterns. It is known that the amplitude of T wave is maximal in lead V3 [18]. In addition, T wave in the precordial leads (<10 mm or <1 mV) is usually greater than that in the limb (<5 mm or 0.5 mV) leads [18]. In our results, only less than 2% in the total patient group (6 in lead II, 5 in lead V3, 5 in lead V4, 1 in lead V5, and 0 in lead V6 among 330 ECGs) and the normal ECG subgroup (2 in lead II, 4 in lead V3, 1 in lead V4, 0 in lead V5, and 0 in lead V6 among 231 normal ECGs) exceeded 0.5 mV in the limb lead or 1 mV in the precordial leads, respectively. Furthermore, the average value of measured T-amp was highest in V3 and that of precordial leads was significantly higher than that of the limb lead (lead II) in one-way analysis of variance and in Post-Hoc Tukey's test (*p*<0.001). It could signify that the extracted values are reliable and could be used for further analysis.

Similar to the findings of previous research conducted in clinical settings, T-wave-based features had no clear relationship with serum potassium level. According to a prior study, changes in ECG pattern, which are suggestive of hyperkalemia, were noted in only 46% of patients whose potassium level ranged between 6 and 9.3 mEq/L [19]. Several other case reports also supported the finding that significant ECG changes are not related to markedly elevated potassium levels [20, 21]. In addition, patients we meet in daily practice have diverse confounding factors, such as medications, comorbidities, and demographics. Because these confounding factors might alter ECG data, they make discovering patterns that are clearly associated with serum potassium level more difficult. Because of this, the sensitivities of detecting hyperkalemia by two physicians in an emergency department were very low at 0.43 and 0.34 [13].

In the subgroup analysis of normal ECG or otherwise normal ECG, the results showed the same pattern of no correlation between T-wave features and serum potassium level. We believe that this cannot be due to contamination by abnormal ECGs. Although we selectively applied the serum potassium level determination model to normal ECG, it might be difficult to get reliable performance.

Zachi et al. also attempted to estimate serum potassium level based on T-wave features, and the estimation performance decreased when the developed model was applied to another independent test group [16]. Higher performance was observed when the estimation model was applied to the patients who were used for model development but at different time points. This finding suggests that unique ECG patterns are caused by different characteristics of each patient; therefore, a personalized model rather than one that can be generally applied should be developed.

The deep learning approach could be an alternative model. Deep learning, a machine learning model, has emerged as the most popular design in various applications, including computer vision and natural language processing. In particular, convolutional neural networks can act as feature extractors from data even in the absence of prior knowledge of the domain [22], and the recurrent neural network model identifies temporal dependencies in time series problems [22]. Feature extraction and time dependencies can be effectively captured by combining both models. If the deep learning-based model is used, more diverse and complex features can be extracted from ECG.

Our study has some limitations. First, T-amp and T-right slope were measured manually rather than automatically because there have been issues with determining the end of the T-wave. Because the end of the T-wave transits very slowly from around the signal, locating the end of the T-wave is one of the most challenging issues in the evaluation of the ECG waveform [23–25]. By having two independent evaluators perform the measurements and then using only accordant results, we attempted to ensure the reliability of our results. Second, the length of the waveform used in the study was short (about 3 seconds). Our data might be relatively limited and less tolerant of noise or artifacts. Finally, we did not consider other ECG patterns, such as QRS widening or P wave flattening, which can also be observed in hyperkalemia. However, T-wave change is known as the most representative and earliest sign of hyperkalemia.

## 5. Conclusions

As shown by findings from previous research, our study also showed that T-wave-based features were not correlated with serum potassium levels in real-world clinical practice in the Korean population; even in the normal ECG subgroup, we could not detect any correlation. Therefore, the use of these features in the estimation of serum potassium level in real clinical practice is very limited.

## Figures and Tables

**Table 1:** Baseline characteristics of the subjects.

Variable	Total	Normal ECG subgroup*∗*
No. of patients, n	330	231
Age (years), mean±SD	47.6±17.2	45.9±15.1
Male sex, n (%)	140 (42.4)	89 (38.5)
Potassium level (mmol/L), mean±SD	4.17±0.39	4.35±0.59
No. of normal sinus rhythms, n (%)	254 (77.0)	191 (82.7)
No. of normal ECG, n (%)	231 (70.0)	231 (100.0)
Amplitude of T-wave (mV), mean±SD		
Lead II	0.23±0.11	0.24±0.10
Lead V3	0.43±0.24	0.42±0.23
Lead V4	0.40±0.22	0.39±0.20
Lead V5	0.38±0.19	0.37±0.17
Lead V6	0.32±0.16	0.32±0.14
Gradient of T-wave (mV/s), mean±SD		
Lead II	-3.06±1.51	-3.1±1.39
Lead V3	-5.13±3.18	-5.02±3.07
Lead V4	-5.02±3.13	-4.93±2.88
Lead V5	-4.81±2.78	-4.78±2.49
Lead V6	-4.21±2.30	-4.24±2.07

SD: standard deviation; no.: number; ECG: electrocardiogram. *∗*Normal ECG or otherwise normal ECG.

**Table 2:** Pearson correlation coefficients between T-wave features from each lead and serum potassium level.

Group	Feature	II	V3	V4	V5	V6	Pt
Total	T-amp	0.08	0.18	0.17	0.17	0.13	0.19
	T-right slope	-0.02	-0.12	-0.10	-0.12	-0.07	-0.11
	T-norm	-0.14	-0.19	-0.21	-0.21	-0.18	-0.22

Normal ECG subgroup*∗*	T-amp	0.05	0.15	0.15	0.15	0.11	0.16
	T-right slope	-0.00	-0.13	-0.10	-0.11	-0.07	-0.12
	T-norm	-0.08	-0.15	-0.16	-0.16	-0.13	-0.17

T-amp: amplitude; T-right slope: right slope of the T-wave; T-norm: normalized feature. *∗*Normal ECG or otherwise normal ECG.

## Data Availability

Data of the measurement of T-wave-based features used to support the findings of this study are included in the supplementary information file.

## Supplementary Materials

Supplementary 1 Data S1. Values of T-wave-based features and the corresponding serum potassium values.

Supplementary 2 Table S1. The detailed interpretation lists of otherwise normal ECG and the count per interpretation.
