# Supplementary material for: Quantitative Evaluation of the Relationship between T-Wave-Based Features and Serum Potassium Level in Real-World Clinical Practice
Source: Biomed Res Int. 2018 Dec 18;2018:3054316. doi: 10.1155/2018/3054316 (PMC6312577; doi:10.1155/2018/3054316)
Supplement: Supplementary 2 — Table S1. The detailed interpretation lists of otherwise normal ECG and the count per interpretation. [file 3054316.f2.pdf]

Table S1. The detailed interpretation lists of otherwise normal ECG and its count per each interpretation

Interpretation	Count
Sinus bradycardia/Otherwise normal ECG	29
Sinus rhythm with 1st degree A-V block/Otherwise normal ECG	4
Sinus tachycardia/Otherwise normal ECG	4
Sinus tachycardia with 1st degree A-V block/Otherwise normal ECG	1
Sinus bradycardia with 1st degree A-V block/Otherwise normal ECG	1
Sinus bradycardia with Premature atrial complexes/Otherwise normal ECG	1
